# Mechanisms Underlying T Cell Immunosenescence: Aging and Cytomegalovirus Infection

**DOI:** 10.3389/fmicb.2016.02111

**Published:** 2016-12-27

**Authors:** Wenjuan Tu, Sudha Rao

**Affiliations:** Faculty of ESTeM, Health Research Institute, University of CanberraCanberra, ACT, Australia

**Keywords:** immunosenescence, naive and memory T cells, aging, HCMV infection, epigenetic regulation, vaccination

## Abstract

The ability of the human immune system to protect against infectious disease declines with age and efficacy of vaccination reduces significantly in the elderly. Aging of the immune system, also termed as immunosenescence, involves many changes in human T cell immunity that is characterized by a loss in naïve T cell population and an increase in highly differentiated CD28- memory T cell subset. There is extensive data showing that latent persistent human cytomegalovirus (HCMV) infection is also associated with age-related immune dysfunction in the T cells, which might enhance immunosenescence. Understanding the molecular mechanisms underlying age-related and HCMV-related immunosenescence is critical for the development of effective age-targeted vaccines and immunotherapies. In this review, we will address the role of both aging and HCMV infection that contribute to the T cell senescence and discuss the potential molecular mechanisms in aged T cells.

## The aging immune system

The human immune system must fight diverse pathogens and provide sufficient host protection throughout life. Memory T cells, which differentiate from naïve T cells upon primary antigenic stimulation and enable a rapid and robust response to previously encountered pathogens, are key players in adaptive immunity. The generation and maintenance of pathogen-specific memory T cells is crucial for life-long immune protection and effective vaccination (Farber et al., 2014). However, profound changes occur in the human immune system over time, known as immunosenescence. These age-related changes contribute to decreased immune protection against infections and diminished responses to vaccination in the elderly. Changes in T cell immunity appear to be have the most impact (Miller, 1996; Cambier, 2005).

Although T cell numbers remain more or less constant over the human lifespan, pronounced age-associated changes occur in T cell composition (naïve vs. memory T cell subsets). It is well accepted that the functional naïve T cell output decreases after puberty due to thymic involution, resulting in increased homeostatic proliferation of existing naïve T cells and eventually phenotypic conversion of naïve T cells into virtual memory cells (Nikolich-Žugich, 2008, 2014; Goronzy et al., 2015; Jacomet et al., 2015). In contrast to the shrinking naïve compartment and its impaired ability to activate and differentiate with age, the proportion of memory T cells increases during early life, remains stable throughout adulthood, but starts to show senescent changes after about 65 years (Farber et al., 2014). In humans, circulating memory T cells can be subdivided into two major phenotypically and functionally distinct populations: central memory T cells (T_CM_; CD45RA^−^CCR7^+^CD62L^+^), which are largely confined to secondary lymphoid tissues, and effector memory T cells (T_EM_; CD45RA^−^CCR7^−^CD62L^−^), which can traffic to multiple peripheral compartments (Sallusto et al., 1999; Mueller et al., 2013; Farber et al., 2014). T_CM_ cells are enriched for CD4^+^ T cells, while T_EM_ cells are predominantly CD8^+^ T cells in human blood (Moro-García et al., 2013).

One of the most prominent T cell changes to occur with age is the loss of the co-stimulatory molecule CD28 and the progressive accumulation of highly differentiated CD28^−^ T_EM_ cells (CD45RA^+^CD28^−^CCR7^−^CD62L^−^), mainly in the CD8^+^ T cell population (Koch et al., 2008). These cells are characterized by decreased proliferative capacity, shortened telomeres, a reduced TCR repertoire, and enhanced cytotoxic activity. As CD28 is crucial for complete T cell activation, CD28 loss is associated with increased susceptibility to infections and a weakened immune response to vaccination in older people (Saurwein-Teissl et al., 2002; Almanzar et al., 2005; Sansoni et al., 2008; Moro-García et al., 2013). However, CD28^−^ T cells are not anergic, so they might also play a role in tissue-mediated immunity (CD8^+^CD28^−^ T cells) (Flavell et al., 2013) and cytomegalovirus (CMV) infection control (CD4^+^CD28^−^ T cells) (Moro-García et al., 2013). Further studies to explore the generation and maintenance of CD28^−^ T cells, especially in different disease states, will help establish their immune function and enhance our understanding of human T cell aging.

It is thought that the memory T cells generated in youth are well preserved and remain strongly protective over decades (Hammarlund et al., 2003, 2005), while T cell memory responses first derived in old age are severely impaired (Haynes et al., 2003; Weinberger et al., 2008; Nikolich-Žugich and Rudd, 2010; Valkenburg et al., 2012). Therefore, age-targeted vaccines and immunotherapies are required.

The ability to generate protective immune responses largely depends on the generation and maintenance of a diverse and well-balanced T cell repertoire. Several studies have shown contraction in T cell diversity corresponding to a shrinkage in the naïve T cell compartment in elderly individuals due to thymic involution (Naylor et al., 2005; Britanova et al., 2014). However, these studies do not take the dramatic influence of latent persistent infection into account, particularly CMV infection, which is known to be associated with age-related alterations in the T cell pool and function. Recent evidence suggests that homeostatic proliferation maintains the naïve CD4^+^ T cell compartment and its diverse repertoire, but not naïve CD8^+^ T cells, in CMV-negative individuals. A decline in naïve CD4^+^ T cell subsets occurs in the presence of CMV, but there is no depletion of naïve CD8^+^ T cells (Wertheimer et al., 2014). In principle, thymic involution should have an equal impact on both CD4^+^ and CD8^+^ T cells. Therefore, the differences seen between the two subsets suggest that shrinkage of the naïve CD8^+^ T cell pool is more likely to be due to increased development of virtual memory T cells (which are well characterized in murine models Sprent and Surh, 2011; Renkema et al., 2014) than the defective regeneration ability of an aged thymus. Moreover, unprimed “innate/memory-like” CD8^+^ T cells have recently been identified in humans (Jacomet et al., 2015). Taken together, these data imply that thymic involution might be less important for maintaining T cell diversity than previously thought. Characterization of the epigenetic signatures and transcriptional profiles of these virtual memory T cells will help explain how antigen-inexperienced T cells acquire the memory phenotype and how they contribute to the aging immune system.

Given that polyfunctional T cells confer a more effective immune response to infections (Yamamoto et al., 2009; Boyd et al., 2015; Snyder et al., 2016), it is important to investigate the impact of aging on T cell polyfunctionality. Van Epps et al. demonstrated enhanced polyfunctionality in CD8^+^ T_EM_ cell subsets in older individuals, but both CD4^+^ and CD8^+^ T_CM_ cells exhibited an age-associated decline in polyfunctionality (Van Epps et al., 2014). However, the CMV-seropositive reaction was again not examined, and the possibility that increased polyfunctional CD8^+^ T_EM_ cells may be due to repeated antigenic stimulation could not, therefore, be excluded.

Despite intensive studies of T cells providing some insights into immune system aging, they have a number of limitations that need to be taken into consideration in future investigations. First, most of our current knowledge on T cell aging is based on studies of circulating peripheral blood T cells, which only represent 2% of the total T cell pool (Farber et al., 2014). Circulating memory T cells predominantly reside in tissues other than the blood (Mueller et al., 2013). In addition, a non-circulating subset of memory T cells has recently been identified and designated tissue-resident memory T cells (T_RM_; CD45RA^−^CCR7^−^CD62L^−^CD69^+^), which permanently reside in peripheral tissues and enhance local immunity during infections (Mueller et al., 2013; Farber et al., 2014; Clark, 2015). However, age-related changes in naïve and memory T cells in distinct human organs have only rarely been studied (Lazuardi et al., 2005; Herndler-Brandstetter et al., 2012; Sathaliyawala et al., 2013). Second, most studies are limited to the total CD4^+^ T cell pool with only a few studies conducted in Th1 and Th2 CD4^+^ T cell subsets, and even less is known about the impact of aging on other lineages, such as Th17 cells (Huang et al., 2008; Tesar et al., 2009; Lee et al., 2011; Lim et al., 2014), regulatory T cells (Tregs) (Fessler et al., 2013; Garg et al., 2014), and Tfh cells (Winkler and Waisman, 2014; Zhou et al., 2014). Finally, human memory T cells are generated and maintained in the context of exposure to diverse viral infections throughout life, particularly CMV infection (over 90% of young people in developing countries) (Arens et al., 2015). It is well-known that CMV plays an important role in human memory T cell function with aging. Therefore, distinguishing CMV seropositive individuals from others is important to provide a more accurate understanding of age-related memory T cell immunity. The enormous impact of CMV infection on T cell function with aging is further discussed below.

## Molecular mechanisms of T cell senescence

Although the impact of aging on T cells is relatively well described at the cellular level, the molecular mechanisms of aging are rather less well understood. There is growing evidence that altered transcription (Chen et al., 2013) and epigenetic regulation (Ponnappan and Ponnappan, 2011) are involved in T cell senescence.

A major feature of immunological memory is the ability of T cells to remember their previous transcriptional profile and propagate their gene expression pattern to progeny, termed “adaptive transcriptional memory” (Turner, 2003; Kundu and Peterson, 2009). In transcriptional or epigenetic memory, genes are marked with active epigenetic signatures that are maintained throughout multiple cell divisions. These marks facilitate more efficient and robust transcription upon encountering a secondary stimulus (Zediak et al., 2011).

Chen et al. provided an excellent overview of the impact of aging on genome-wide transcriptional profiles in CD4^+^ and CD8^+^ T cells (Chen et al., 2013). For example, CD8^+^ T cells (Fann et al., 2005), and to a lesser degree CD4^+^ T cells (Czesnikiewicz-Guzik et al., 2008), decrease CD28 gene expression with age, with CD28^+^ and CD28^−^ CD8^+^ memory T cells showing different transcriptional profiles. There was elevated expression of several transcription factors including T-bet, eomesodermin (EMOES), and MYC (Fann et al., 2005) in CD28^−^ memory T cells. T-bet and EMOES play crucial roles in the acquisition of T cell effector functions and memory development in CD8^+^ T cells and regulate IFN-γ, granzyme B, and perforin expression (Angelosanto and Wherry, 2010), suggesting that they might critically mediate the enhanced cytotoxicity of CD28^−^CD8^+^ memory T cells. In addition, mammalian target of rapamycin (mTOR) has been shown to modulate memory CD8^+^ T cell formation by regulating T-bet and EMOES gene expression. mTOR inhibition promotes T cell memory by repressing T-bet and inducing EOMES (Rao et al., 2010). Remarkably, the mTOR inhibitor RAD001 has recently been reported to improve immune responses to influenza vaccination in elderly volunteers, but the underlying mechanism remains obscure (Mannick et al., 2014). Two possible mechanisms might explain this observation. First, T-bet expression is higher in terminally differentiated T_EM_ cells (Joshi et al., 2011), while EOMES is highly expressed in persistent T_CM_ cells (Banerjee et al., 2010). Therefore, RAD001 might restrain the conversion of memory T cells to a highly differentiated phenotype, such as CD28^−^ T_EM_ cells, by reducing T-bet expression in the elderly. As a consequence, T cell senescence is suppressed and the immunological function of memory T cells enhanced. Second, T-bet is also the master regulator of Th1 cell differentiation but is inhibitory in Th2 and Tfh cell lineage development (Lazarevic et al., 2013). Zang et al. found a Th1 CD4^+^ subset shift and reduced Th2/Tfh cell differentiation in aged mice (Zhang et al., 2014). Hence, the decrease in T-bet caused by RAD001 might enhance Th2 and/or Tfh CD4^+^ cell development, which is crucial for B cell immunity and antibody production. This latter hypothesis is further supported by clinical observations of an increased antibody response in elderly volunteers receiving the influenza vaccination (Mannick et al., 2014). Further studies of cellular and molecular changes occurring in these different subsets are needed.

Numerous studies have shown that epigenetic mechanisms including DNA methylation, histone modifications, chromatin structure alterations, and microRNA regulation play an essential role in transcriptional memory by regulating gene expression in memory T cells (Gibney and Nolan, 2010). It is well-known that genome-wide decreases in the methylation of repetitive elements (particularly Alu sequences) and gene coding regions occur during aging (Bollati et al., 2009; Heyn et al., 2012; Salpea et al., 2012). These age-dependent DNA methylation changes also occur in immune-specific genes that contribute to T cell senescence (Shanley et al., 2009; Fernández-Morera et al., 2010; Hongdong et al., 2015; Tserel et al., 2015).

Although the age-associated DNA methylation signatures that mark immune responsive genes are well documented, little is known about the contribution of site-specific histone modifications to immunosenescence. Histone acetylation, methylation, phosphorylation, and ubiquitination all regulate transcription by altering chromatin accessibility (Strahl and Allis, 2000; Kouzarides, 2007). Early epigenetic studies in rat livers showed significant decreases in histone acetylation (H3K9ac) and H3 phosphorylation (H3S10ph) contributing to age-associated decreases in transcription (Kawakami et al., 2008). More recently, Sidler et al. demonstrated profound age-dependent changes in gene expression accompanied by decreases in DNA methylation, H3K9me3, and H4K16ac in the rat spleen and thymus, suggesting increased chromatin instability with age. Moreover, several transcription factors (BCL6, MYC, TCF7, and ETS1) with decreased expression in the thymus and increased expression in the spleen are thought to be associated with immunosenescence (Sidler et al., 2013). Since these transcription factors play crucial roles in homeostatic proliferation, differentiation, and activation in T lymphocytes (Muthusamy et al., 1995; Angelosanto and Wherry, 2010), their opposing transcriptional profiles in the thymus and spleen might contribute to the distinct senescence phenotype observed during aging, i.e., the reduced thymic naïve T cell output and increased homeostatic proliferation of peripheral T cells. Chromatin immunoprecipitation (ChIP) studies to identify genome-wide epigenetic signatures with characterization of chromatin accessibility in these regions might provide insights into the epigenetic regulation of immunosenescence.

Several recent studies have explored microRNA regulation in T cell aging. For example, miR-92a expression significantly declined in CD8^+^ T cells during aging, which also correlated with reduced naïve CD8^+^ T cell numbers. It has also been suggested that the progressive decline in miR-92a expression may be associated with a reduced naïve T cell repertoire due to repeated stimulus exposure (Ohyashiki et al., 2011). Moreover, an age-dependent decline in miR-181a, an intrinsic regulator of TCR signaling (Li et al., 2007), was observed in aged CD4^+^ T cells (Li et al., 2012). miR-181a loss increased DUSP6 activity, which eventually impaired TCR sensitivity in elderly CD4^+^ T cells (Li et al., 2012). Compared to CD8^+^CD28^+^ T cells, highly differentiated CD8^+^CD28^−^ T cells overexpressed miR-24, which was associated with downregulation of the histone variant H2AX and consequent impairment of the response to DNA damage and increased susceptibility to apoptosis in CD8^+^CD28^−^ T cells (Brunner et al., 2012). Taken together, these studies suggest that microRNAs are causative in immunosenescence and might be useful biomarkers of T cell aging. However, a systematic survey of microRNAs, their direct targets, and function in immunosenescence are needed. Comprehensive studies in well-defined human T cell subsets in both health and disease will enhance our understanding of the molecular mechanisms responsible for age-related T cell impairment so that effective interventional strategies against age-associated diseases can be developed.

Apart from genetic/epigenetic inheritance, lifestyle and environmental factors, such as nutritional status and metabolism also have a significant impact on the human immune system and lifespan (Fuente, 2014). There is good evidence that diets that contain particular nutrient or non-nutrient components can significantly enhance immune function in the elderly (Lesourd et al., 1998; Pae et al., 2011; Maijó et al., 2014). For example, dietary manipulation of the availability of methyl donors can extend and maintain immune function by epigenetically modulating methylation in mice (Miller et al., 2005). The mTOR pathway has provided a focus for understanding how metabolism regulates T cell immunity: upon growth factor stimulation, the PI3K/Akt/mTOR pathway is activated that, in turn, promotes nutrient uptake, enhances many metabolic activities, and results protein biosynthesis in T cells (Jones and Thompson, 2007). The mTOR inhibitor rapamycin promotes CD8^+^ T cell memory development (Araki et al., 2009), which might be due to metabolic changes in CD8^+^ T cells (Peng et al., 2002; Sipula et al., 2006; Brown et al., 2007). Moreover, mTOR plays a crucial role in the regulation of effector and regulatory T cell lineage commitment (Delgoffe et al., 2009), since it not only acts as a regulator of translation but also functions as an intracellular energy sensor that positively and directly regulates mitochondrial respiration (Desai et al., 2002; Ramanathan and Schreiber, 2009). A study of energy metabolism has shown that highly differentiated CD8^+^CD28^−^ T cells utilize different metabolic strategies than normal CD8^+^ memory T cells (Henson et al., 2014); in contrast to the quiescent catabolic metabolism in CD8^+^CD28^+^ memory T cells, CD8^+^CD28^−^ T cells have lower mitochondrial mass and respiration, high basal glycolysis levels, and impaired metabolism due to increased ROS levels (Henson et al., 2014). Further, CD8^+^CD28^−^ T cells favor the use of the cytosolic glycolytic pathway over mitochondrial respiration for energy production. Aerobic glycolysis is a critical metabolic pathway required for the production of the effector cytokine IFN-γ, consistent with the enhanced cytotoxic activity of CD8^+^CD28^−^ T cells (Chang et al., 2013; Gubser et al., 2013). As mitochondrial function is compromised during aging (Ron-harel et al., 2015), a switch to energy generation by glycolysis may produce a population of CD8^+^CD28^−^ T cells with the metabolic advantage to outcompete other T cell subsets competing for nutrients and factors required for survival and function. These data highlight the important role played by nutrient status and metabolic regulation in enhancing T cell immunity and might be a promising approach to promote healthy aging through nutritional and metabolic interventions.

## Human CMV infection, memory inflation, and immunosenescence

Cytomegalovirus (CMV) is an ubiquitous β-herpesvirus with a double-stranded DNA genome that has co-evolved with humans over millions of years (Sinclair and Sissons, 2006; Gibson, 2008). Human CMV (HCMV) is a prevalent human pathogen, infecting 40–100% of world's population. CMV has the capacity to induce both lytic and latent infections to establish lifelong persistence in human hosts following primary infection (Cannon, 2009; Ludwig and Hengel, 2009; Cannon et al., 2010). In lytic infection, HCMV undergoes temporally active replication that can be divided into immediate early (IE), early (E), and late (L) phases. The most crucial viral lytic gene products are viral IE genes, which control subsequent viral gene expression and virus replication. Thus, these major IE gene products essentially determine the HCMV infection states: latency (IE genes suppressed) or reactivation (IE genes expressed) (Sinclair and Sissons, 2006; Paulus and Nevels, 2009). The latent phase of HCMV infection is characterized by viral quiescence, in which the viral genome is maintained as an extra-chromosomal plasmid in the absence of detectable production of infectious virions but able to reactivate to specific stimuli (Goodrum et al., 2002; Reeves et al., 2005; Sinclair and Sissons, 2006). Despite infecting a broad range of host cells, HCMV usually establishes viral latency at specific cellular sites, predominantly CD34^+^ progenitors and myeloid lineage cells (Hahn et al., 1998; Goodrum et al., 2002).

Primary HCMV infection elicits extensive innate and adaptive immune responses. Thus, to escape host antiviral responses, HCMV has developed diverse immune evasion strategies to alter host immune recognition during both lytic and latent infection. In particular, HCMV restricts major histocompatibility complex (MHC) class I and II antigen presentation, which allows the virus to survive, disseminate, and persist in infected individuals (Noriega et al., 2012). In immunocompetent individuals, both primary and lifelong persistent HCMV infections generally remain subclinical and well controlled by the host immune system. T cell responses are particularly important for controlling viral latency in infected individuals (Hanley and Bollard, 2014; Klenerman and Oxenius, 2016). During the early phases of primary infection, the initial T cell response to HCMV is dominated by circulating HCMV-specific CD4^+^ T cells that produce the Th1 cytokines IFN-γ and TNF-α (Rentenaar et al., 2000; Gamadia et al., 2003). Highly cytotoxic HCMV-specific CD8^+^ T cells can be detected in the blood several days after the initial CD4^+^ T cell response, and they are maintained with an effector phenotype during the latent phase to prevent HCMV reactivation and to protect the host from re-infection (Polić et al., 1998; Kuijpers et al., 2003; Mackus et al., 2003). In addition, the humoral response to HCMV also contributes to controlling the viral load and preventing primary infection in humans (Gerna et al., 2008; Genini et al., 2011; Jackson et al., 2011; Alonso Arias et al., 2013). After primary infection, B cells are activated by antigens with the help of HCMV-specific CD4^+^ T cells, mainly Th2 cells, leading to the production of antibodies specific for a number of HCMV proteins (Wang and Shenk, 2005; Gerna et al., 2008; Macagno et al., 2010; Genini et al., 2011). These HCMV-specific antibodies can block viral dissemination and control the infection by neutralizing extracellular virions, a mechanism that is particularly important for protecting the fetus from congenital HCMV infection (Fowler et al., 1992; Revello and Gerna, 2002; Schleiss, 2013).

However, long-term HCMV persistence has a profound impact on the immune system's composition and function, even in healthy HCMV-infected individuals, especially with respect to CD8^+^ T cells. One hallmark of latent HCMV infection is the progressive and substantial expansion of HCMV-specific memory CD8^+^ T cells over time, with HCMV-specific memory CD4^+^ T cells accumulating to a lesser extent (Klenerman and Oxenius, 2016; Weltevrede et al., 2016). This accumulation of HCMV-specific memory T cells during viral persistence is termed “memory inflation,” first defined in the mouse CMV (MCMV) infection model (Karrer et al., 2003). HCMV-specific memory T cells tend to gradually increase in number with age: in HCMV-infected elderly individuals, the CD8^+^ T cell response to HCMV antigens occupies nearly 50% of the entire memory CD8^+^ T cell compartment in peripheral blood, while approximately 30% of total circulating CD4^+^ T cells can be HCMV responsive (Sylwester et al., 2005; Pourgheysari et al., 2007; Li et al., 2014).

Human cytomegalovirus (HCMV) persistence is thought to be a driver of immunosenescence in humans (Koch et al., 2007). The majority of HCMV-specific inflationary T cells are T_EM_ cells with the typical age-related senescent T cell phenotype. These terminally differentiated HCMV-specific T cells generally acquire CD57 and CD45RA expression but lack CD28 and CCR7 expression (CD45RA^+^CD57^+^CD28^−^CCR7^−^) (Gamadia et al., 2001; Appay et al., 2002; Kuijpers et al., 2003). It is widely accepted that late-stage differentiated CD28^−^ T cells are a major characteristic of T cell aging, suggesting that persistent HCMV infection is associated with immunosenescence. This is further supported by the fact that the large population of HCMV-specific CD8^+^CD28^−^ T_EM_ cells that usually accumulate during HCMV persistence are absent in HCMV-seronegative elderly individuals, even those infected with other persistent herpes viruses (Chidrawar et al., 2009; Derhovanessian et al., 2010). Several studies have shown that acute viral infections generate polyfunctional memory CD8^+^ T cells with re-expressed CD45RA (Precopio et al., 2007; Miller et al., 2008; Akondy et al., 2009). Live yellow fever vaccine (YF-17D)-specific memory CD8^+^ T cells exhibited a terminally differentiated CD45RA^+^CCR7^−^ phenotype but remained polyfunctional with robust proliferative potential 5–10 years post vaccination, which may suggest that re-expressed CD45RA biomarks highly functional memory T cells after acute viral infections rather than senescent T cells (Akondy et al., 2009). However, these YF-17D-specific memory cells were not “true” late-stage differentiated CD28^−^ T cell subsets but CD27^+^CD28^+^ cells. Therefore, the use of CD45RA alone as a marker of T cell differentiation and function is questionable. In fact, the CD45RA^+^CCR7^−^CD8^+^ subtype represented resting memory T cells that could be re-activated upon antigenic stimulation. It has also been shown that CD45RA re-expression on memory T cells can occur in the absence of further antigenic stimulation, which is indicative of the time elapsed since previous viral infection rather than the functional potential of CD8^+^ T cells (Carrasco et al., 2006). In addition, CD45RA^+^CD8^+^ memory T cells did not accumulate with age and showed no correlation with the CD28^−^CD8^+^ T_EM_ subset (Mahnke et al., 2013). Thus, CD28 is a more reliable surface marker for the phenotypic and functional definition of human T cell differentiation. In contrast to age-dependent T cell senescence, increased HCMV-specific memory CD8^+^ profiles (up to 10% of the total CD8^+^ T cell memory pool) were observed even in young hosts, suggesting that progressive clonal expansion may lead to immunosenescence at an earlier age in HCMV-infected individuals (Khan et al., 2004; Chidrawar et al., 2009). However, the high frequency of HCMV-responsive T cells may be a “necessary evil” to restrain HCMV re-activation and maintain control of latent HCMV throughout life (Pawelec, 2005).

There is increasing evidence to suggest that memory inflation in HCMV infection is associated with impaired T cell immunity in elderly hosts. Despite the CD8^+^ T cell repertoire being diverse enough to recognize different viral epitopes soon after primary HCMV infection, clonal diversity starts to shrink with age, with a large proportion of the repertoire limited to a few high-avidity clones with a replicative senescent phenotype (Day et al., 2007). In particular, T cell responses specific to an individual immunodominant HCMV epitope [such as 65kDa phosphoprotein (p65) and 55kDa IE protein 1 (IE1)] can comprise over 25% of the total CD8^+^ T cell population in elderly individuals (Khan et al., 2002; Sylwester et al., 2005). Thus, the excess expansion of a single HCMV-specific repertoire in memory inflation may compromise immune protection in response to novel and vaccine antigens by decreasing TCR diversity in the elderly. This renders individuals with only limited virus-specific T cell clones at risk of life-threatening diseases as they get older (Klenerman and Oxenius, 2016). Additionally, memory inflation in latent HCMV infection may also impact the balance between Th1 and Th2 cytokine production by CD4^+^ T cells in aged individuals, shifting it in favor of Th1 responses (Saurwein-Teissl et al., 2002; Pawelec et al., 2005). Most inflationary memory CD4^+^ T cells are IFN γ-producing Th1 cells, which might explain the poor antibody-mediated immune responses seen in the elderly (Rentenaar et al., 2000; Bitmansour et al., 2002; van Leeuwen et al., 2006). Indeed, HCMV-specific CD4^+^ T cells were negatively associated with humoral responses to influenza vaccination (Derhovanessian et al., 2013). However, the precise mechanism underlying memory inflation is not fully understood, the current hypothesis being that continuous repetitive antigen exposure during latent HCMV infection contributes to immune system reshaping and enhances the age-related changes in T cell compartments in older adults (Pawelec et al., 2009; O'Hara et al., 2012).

Decreased levels of naïve T cells are also a hallmark of immunosenescence. Several cross-sectional studies have indicated that HCMV has much less of an impact on the naïve T cell pool than on memory T cells (Mekker et al., 2012; Wertheimer et al., 2014), but HCMV status is significantly associated with changes in naïve CD4^+^ T cells (Wertheimer et al., 2014). Lower levels of naïve CD4^+^ T cells appear to be HCMV-seropositivity dependent rather than age related, suggesting differential effects of aging and HCMV infection on T cell subsets (Wertheimer et al., 2014).

The “immune risk profile” (IRP) was developed as part of a longitudinal study to predict mortality and morbidity in the aged (Wikby et al., 2005, 2008). HCMV seropositivity was identified as one of the immune parameters of the IRP, which also included an inverted CD4/CD8 ratio, accumulation of CD8^+^CD28^−^ T cells, and a lower proportion of naïve cells. However, HCMV infection was detected in very old people, compatible with the extended lifespan observed in studies of individuals at extreme old age in Japan. Plasma HCMV titers were not inversely correlated with the proportion of CD28^+^ T cells in (semi-)supercentenarians (105 years or older), suggesting that HCMV titers might not be a powerful predictor of T cell senescence in successful aging (Arai et al., 2015). Therefore, it remains controversial whether prediction of mortality can be used as a direct indicator of immunosenescence.

Even though pronounced memory inflation is thought to result in the accumulation of dysfunctional CMV-specific T cells, several studies have shown that HCMV-specific CD8^+^ T cells are polyfunctional in young and middle-aged hosts and can produce multiple cytokines and induce strong effector immune responses to staphylococcal enterotoxin B (SEB) (Solana et al., 2012a; Pera et al., 2014; Sansoni et al., 2014; Hassouneh et al., 2016). With increasing age, T cell exhaustion, another form of T cell dysfunction, can arise during chronic viral infections, such as with HIV, in which T cells are constantly stimulated by highly replicating viruses (Wherry, 2011). Typically, exhausted T cells fail to control viral infection as they have effector defects (Wherry, 2011) including loss of proliferative potential, decreased cytotoxicity, impaired ability to secrete cytokines (Frebel et al., 2010; Wherry and Kurachi, 2015), and sustained high expression of several inhibitory receptors (e.g., PD1, KLRG1, and CD57) (Blackburn et al., 2009). Although HCMV-specific CD8^+^ T cells also have low proliferative capacity and express senescence markers, such as KLRG1 and CD57 (Vieira Braga et al., 2015), they are not exhausted as they are still highly cytotoxic and produce Th1 cytokines in response to sporadic viral re-activation (Klenerman and Oxenius, 2016). In addition, molecular profiling of HCMV-specific CD8^+^ T cells has demonstrated that PD-1, an inhibitory receptor associated with T cell dysfunction, is expressed at very low levels in healthy individuals (Vieira Braga et al., 2015). Therefore, persistent HCMV infection does not induce massive exhaustion of the T cell repertoire in most immunocompetent individuals. Indeed, HCMV is rarely reported in elderly individuals, suggesting HCMV-specific T cells may be able to control pathological HCMV re-activation during healthy aging. However, the possibility remains that HCMV infection can eventually drive the functional exhaustion of T cells and their extensive accumulation may accelerate immunosenescence in immunocompromised and immunosuppressed individuals (Papagno et al., 2004; Chou and Effros, 2013; Effros, 2016).

Taken together, HCMV infection in the elderly is implicated in immunosenescence and might have a deleterious impact on host immunity and enhance the aging process. Nevertheless, there remains considerable uncertainty regarding the causative role of CMV in immunosenescence. Although it is well-known that HCMV is a common cause of severe morbidity and mortality in immunocompromised individuals (Reeves and Sinclair, 2008), we cannot exclude the possibility that HCMV might improve the polyfunctionality of CD8^+^ T cells and consequently benefit the host immune system, at least in young healthy individuals. Moreover, it is still unclear whether HCMV re-activation occurs more frequently in the elderly than in younger individuals. Hence, whether expansion of HCMV-specific CD8^+^ T cells over time is really deleterious in old age remains unknown. It also remains to be seen to what extent accumulated dysfunctional inflationary memory T cells cause immunosenescence and how deleterious these cells are on other immune components, such as B cells, γδ T cells, and NK cells. Finally, the host immune system might inefficiently control latent HCMV reinfection during aging, thereby allowing the large-scale expansion of virus-specific T-cell clones and further enhancing the immunosenescent profile (Klenerman and Oxenius, 2016; Weltevrede et al., 2016). Thus, both HCMV status and HCMV control must be taken into account in future studies on immune aging.

## Vaccination in the elderly

Vaccination is the most cost-effective and efficient strategy for improving immune responses and protecting humans from infections and other emerging diseases. However, both the efficacy and effectiveness of vaccination decrease in the elderly (Jefferson et al., 2005; Chen et al., 2009; Boraschi and Italiani, 2014; Haussig et al., 2014). Given the rapidly aging population in both developed and developing countries, improving vaccination efficacy to promote healthy aging is a priority, not only for individual well-being, but also for public health.

In the aged population, infectious diseases are a major cause of morbidity and mortality, mainly because the host immune system cannot generate adequate adaptive immune responses to infections (Chen et al., 2009). As discussed above, aging and latent CMV infection are closely associated with immunosenescence by reshaping the host immune repertoire. This can lead to increased susceptibility to severe infections due to the progressive impairment of innate and adaptive immunity in the elderly. In particular, significantly decreased levels of naïve T cells with a restricted TCR repertoire and expansion of highly differentiated memory T cells that gradually polarize to a specific virus can compromise host immune responses to novel virus vaccine antigens, leading to suboptimal vaccination. In addition, impaired immune responses accompanied by repetitive antigen exposure can result in chronic inflammation in the elderly and are thought to contribute to the defective immune response to vaccination by further enhancing immunosenescence (Franceschi et al., 2000; Freund et al., 2010; Solana et al., 2012b). Therefore, a more advanced understanding of both the cellular and molecular basis of immunosenescence is required to develop efficient vaccinations that better protect elderly individuals from infections and age-related diseases.

## Conclusions and perspective

**Figure 1:**
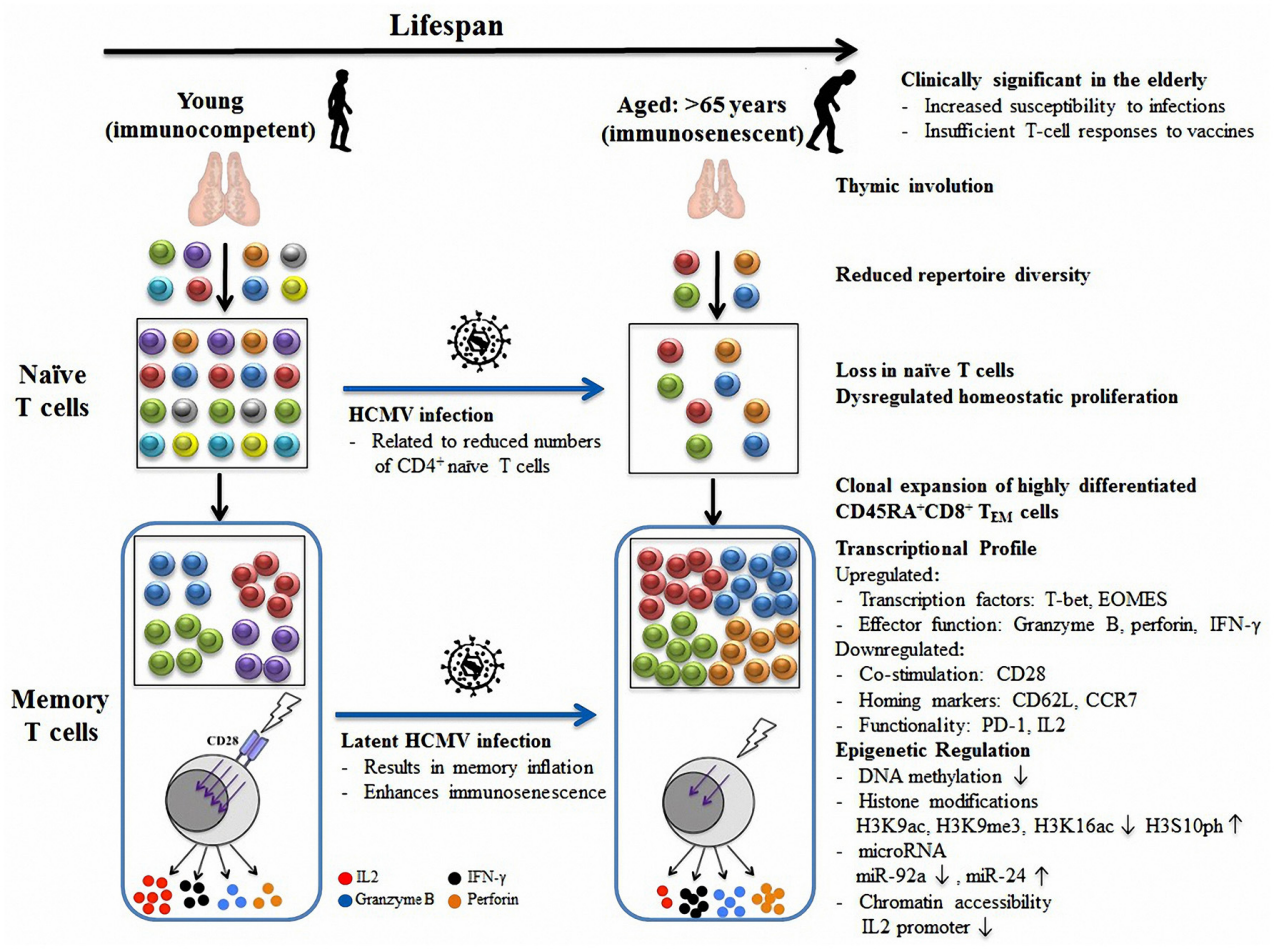
**Model of age- and HCMV-related immunosenescence**. Thymic involution contributes to reduced naïve T cell export and T cell diversity with age. To compensate for inadequate thymic export, existing naïve T cells increase homeostatic turnover. Upon antigen stimulation, a large number of terminally differentiated CD45RA^+^CD8^+^ TEM cells clonally expand in the elderly, which dominate the memory pool and further restrict repertoire diversity. These senescent CD45RA^+^ memory T cells typically have diminished T cell responses to stimulation in the absence of CD28 co-stimulatory signaling pathways and are characterized by a variety of altered transcriptional profiles which are epigenetically regulated (including by DNA methylation, histone modifications, microRNAs, and chromatin remodeling). In addition, HCMV infection can result in CD4^+^ naïve pool depletion and memory inflation, which further accelerate immunosenescence in aged individuals. Together, age and HCMV infection contribute to the overall decline in immune function decline and impair the T cell response to vaccines in the elderly.

The adaptive immune system protects the host from numerous pathogens over the course of human life. However, aging is associated with a quantitative decline in immunity, particularly in T cells, referred to as immunosenescence. Age-related T cell senescence has been attributed to thymic involution, contraction in the T cell repertoire, and accumulation of highly differentiated CD28^−^ T cells. There is growing evidence that latent HCMV infection might accelerate immunosenescence since it causes similar detrimental effects on T cell phenotype and function to those found in age-associated immunological defects. Thus, age-related and HCMV-related immunosenescence - either together or separately - might contribute to increased susceptibility to infectious disease and impaired immune responses to vaccination in the elderly (Figure 1). To prevent severe infections and promote healthy aging, efforts have been made to improve the efficacy of vaccination in the elderly including increased vaccine doses, prime-boost immunization strategies, and the use of adjuvants (Couch et al., 2007; Brown, 2010; Khurana et al., 2011; Dorrington and Bowdish, 2013). Unfortunately, these approaches have yet to significantly improve vaccination outcomes in the older population.

There is also a considerable body of evidence to suggest that epigenetic changes play a crucial role in natural and pathological immune aging (Grolleau-Julius et al., 2009) and a variety of human diseases (Barros and Offenbacher, 2009; Hewagama and Richardson, 2009; Invernizzi, 2009; Wells, 2009). Our unpublished preliminary data suggest that the chromatin accessibility status of certain regulatory elements, in particular that of IL-2, alter over the human lifespan in naïve and memory T cells. This is further supported by findings that epigenetic regulation plays a prominent role in immune responses and age-related differential gene expression of IL-2 (Bruniquel and Schwartz, 2003) and IFN-γ (Yano et al., 2003). A sound knowledge of age-dependent epigenetic gene regulation is essential for optimizing vaccination, perhaps by therapeutically restoring immune function in the elderly. Furthermore, given the crucial role of HCMV in host immune function during aging, an increased understanding of the impact of latent HCMV infection on epigenetic signatures across regulatory elements might provide a novel avenue for overcoming immune defects and improving vaccine efficiency in the elderly. Integrating cellular and epigenetic insights of immunology and virology during aging is essential for the development of age-targeted vaccines and age-dependent immunotherapies that exploit sustained memory responses to pathogens.

## Author contributions

WT participated in drafting the article. SR helped to revise and edit the manuscript and acted as corresponding author.

### Conflict of interest statement

The authors declare that the research was conducted in the absence of any commercial or financial relationships that could be construed as a potential conflict of interest.
